# Parathyroid Cyst: A Case Report of an Uncommon Diagnosis of a Cervical Mass

**DOI:** 10.7759/cureus.9598

**Published:** 2020-08-06

**Authors:** Abdelbassir Ramdani, Marouane Harhar, Tariq Bouhout, Badr Serji, Tijani El Harroudi

**Affiliations:** 1 Surgical Oncology, Mohammed VI University Hospital, Regional Oncology Center, Oujda, MAR

**Keywords:** parathyroid, parathyroid cyst, cervical masses, case report, scare

## Abstract

Parathyroid cysts (PCs) are uncommon entities in clinical practice. The lack of pathognomonic clinical presentation and radiological features of PCs makes preoperative diagnosis unlikely, therefore, most cases are diagnosed intraoperatively or postoperatively at the pathological analysis of the surgical specimen. Treatment of nonfunctional PCs remains controversial and includes fine-needle aspiration, injection of sclerosant, or surgical excision. However, surgical resection still the optimal treatment for functional and larger nonfunctional PCs. We report a case of a 60-year-old female presenting with asymptomatic left-sided cervical swelling diagnosed postoperatively as a nonfunctional PC.

## Introduction

Parathyroid cysts (PCs) are rare entities, representing 0.5%-1% of parathyroid lesions [1]. They are typically classified as functional or nonfunctional, depending on their association with hyperparathyroidism; most PCs are nonfunctional, and they represent approximately 80% of cases [2]. The clinical presentation and radiological findings are nonspecific, making preoperative diagnosis difficult. Treatment options include aspiration, percutaneous sclerosing agent injections, or surgical resection [3]. We report a case of a nonfunctional PC and present the clinicopathological findings and diagnostic challenges, highlighting the importance of including it in the differential diagnosis of cervical masses. The case is reported following the SCARE criteria [4].

## Case presentation

A 60-year-old woman presented with a four-month history of left-sided cervical swelling. No dyspnea, swallowing disturbance, or dysphonia was recorded. Physical examination revealed a soft, painless, and mobile mass on the left side of the neck. Ultrasonography of the neck showed a cystic mass measuring 6 cm in the left thyroid lobe.

**Figure 1:**
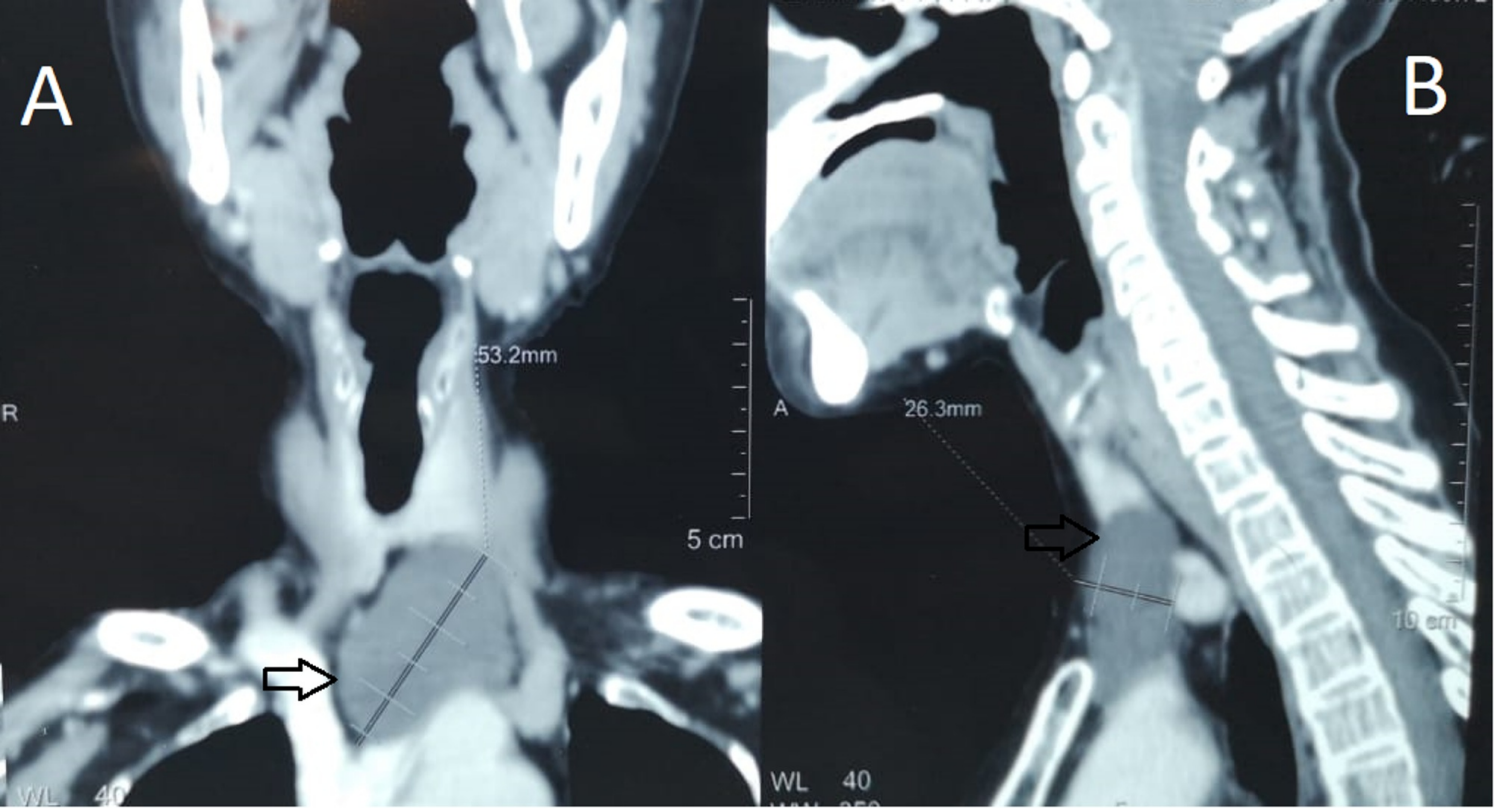
(A and B) Cervical computed tomography scan showing left-sided cervical cystic mass (black arrows) measuring 26x45x53 mm

Thyroid function tests and serum calcium were in the normal range. A cervical CT scan identified a left-sided cystic mass measuring 26 x 45 x 53 mm under the thyroid, and a diagnosis of the fourth branchial cleft cyst was suspected (Figure 1A, B).

**Figure 2:**
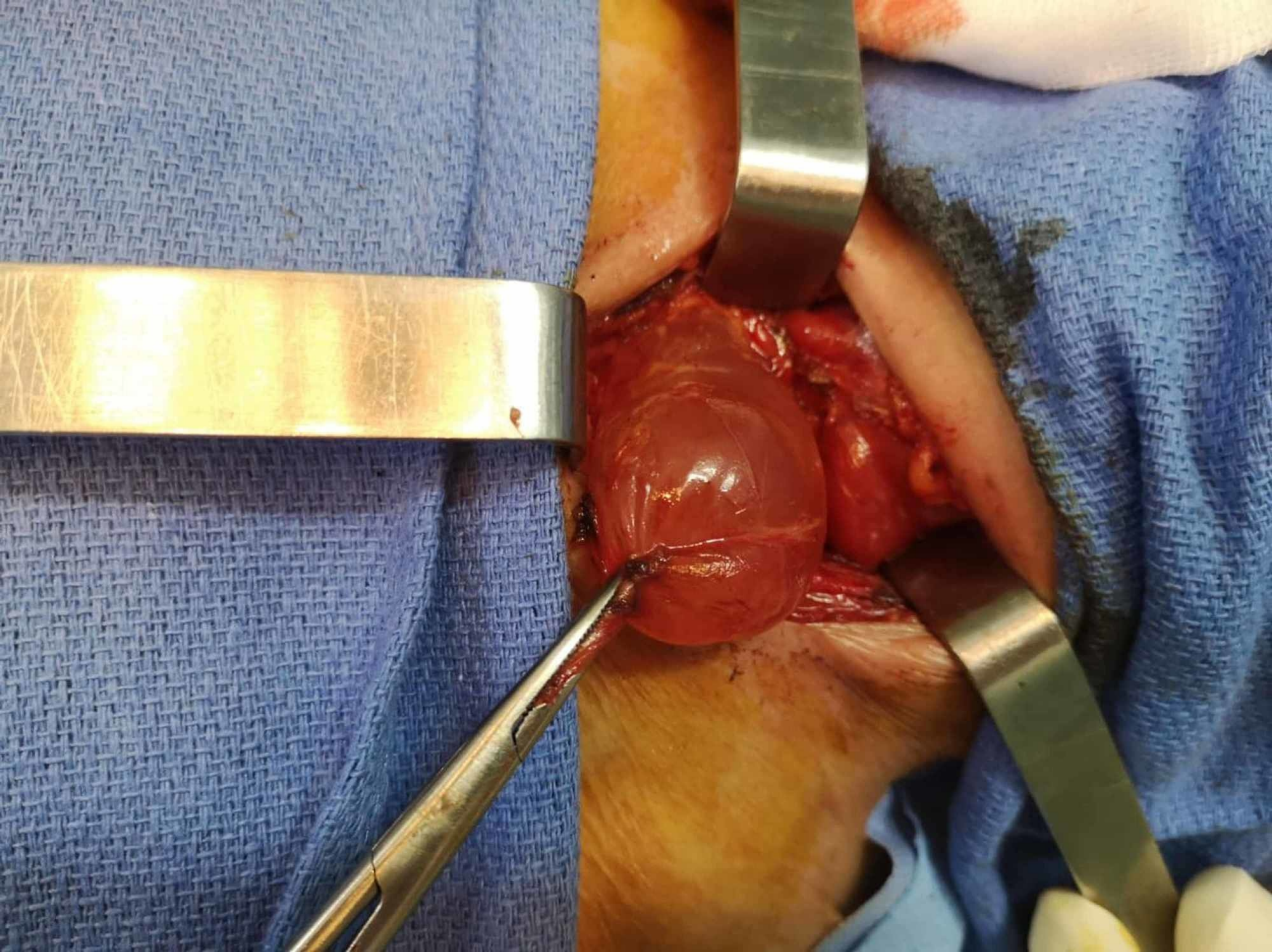
Operative photograph showing a thin-walled cyst released from the left thyroid lobe

**Figure 3:**
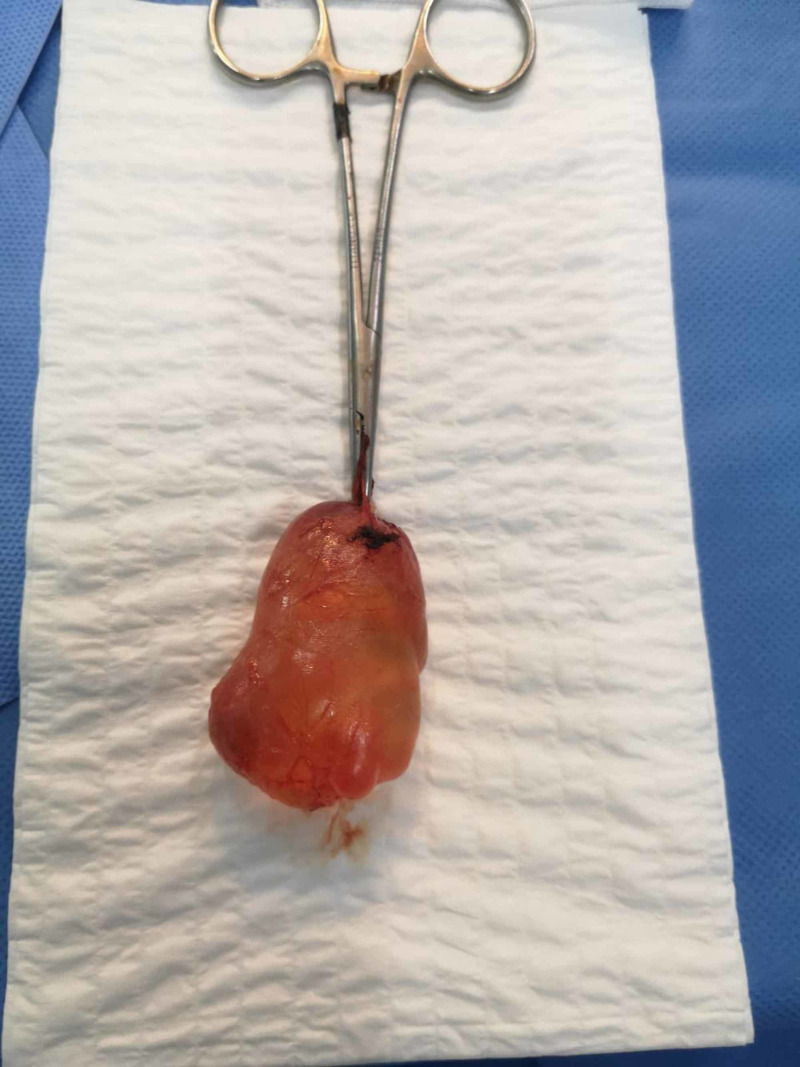
Image showing the resected cyst

Despite the investigations, the diagnosis remained unclear, with the risk of increasing swelling size. The patient underwent exploratory cervicotomy with excision of the cyst. Intraoperatively, the left thyroid gland appeared normal on inspection and palpation. A thin-walled cyst of approximately 5-6 cm was found. This was located between the left thyroid lobe and the trachea but seemed to be separate from both structures (Figures 2, 3).

Histological examination of the specimen showed a 5.5 cm cyst bordered by a heavily abraded squamous epithelium, with the presence of atrophic parathyroid parenchyma. This concluded the diagnosis of a PC.

The patient made an uneventful recovery and was discharged from the hospital on postoperative day two. She remained asymptomatic, with no recurrence at a six-month follow-up.

## Discussion

Described for the first time by Sandström in 1880 [5]. PCs are rare clinical entities, accounting for 0.8%-3.41% of all parathyroid lesions [1]. Goris reported the first surgical resection in 1905 [6]. Only around 360 cases have been reported in the international literature. PCs seem to have a female predilection, with a male/female ratio of 1/1.85. According to Papavramidis et al., they tend to occur more frequently in the fourth and sixth decades [7]. PCs are classified as functional or nonfunctional, and the former type is associated with clinical hyperparathyroidism. Most PCs are nonfunctional, and they represent approximately 80% of cases [2]. The most frequent location remains the left inferior parathyroid gland; however, PCs can extend from the angle of the mandible to the mediastinum [7]. They generally manifest as asymptomatic masses; according to Alvi et al. and Coates et al., larger cysts bring about compressive symptoms, such as dysphagia, odynophagia, dyspnea, and dysphonia [8,9].

The preoperative diagnosis of PCs is challenging due to their heterogeneous clinical presentations and nonspecific radiological findings. Cervical ultrasonography is considered the first-line imaging modality as part of the diagnostic workup; although it cannot distinguish between a PC and other cervical masses, ultrasonography may reveal the cystic nature and dimensions of the mass [10,11].

CT and MRI are used to determine the exact location of the lesion and the relationship with surrounding tissues and adjacent vascular structures. The definitive diagnosis is established by aspiration of cystic liquid, biochemical testing of the sample, and identifying parathormone (PTH) [2]. In most previous studies, it has been reported that the aspiration of clear and colorless fluid suggests PC, and fluid PTH level analysis has been recommended for diagnosis. The PTH level in the cyst fluid is higher than the serum PTH level in both functional and nonfunctional cysts [7]. Fine-needle aspiration was not performed in our case as the diagnosis of PC was not suspected clinically or radiologically. Moreover, from the radiological analysis, the cystic lesion was first considered to be a fourth branchial cleft cyst; however, after surgical resection, it was histopathologically confirmed as a nonfunctional PC. Multiple differential diagnoses of PCs should be considered, such as thyroid goiter, thyroid cyst, thymic cyst, thyroid adenoma, thyroglossal duct cyst, and branchial cleft cyst [2,12].

Treatment of PCs includes simple aspiration, percutaneous sclerosing agent injections, and surgical resection [3]. Ultrasound-guided aspiration can be used alone as an initial treatment for small, nonfunctional, symptomatic PCs with diameters less than 2.5 cm; in the case of recurrence, surgical excision is recommended as the definitive treatment. The disadvantage of this therapeutic approach is the recurrence of the cyst with a very variable success rate even after multiple aspirations, Ippolito et al. reported that four patients had a recurrence among 14 individuals who underwent fine-needle aspiration; however, Sung et al. noted a recurrence rate of 66% in 12 patients treated with fine-needle aspiration [13,14]. Sclerosing agent injections are not recommended because they can cause serious complications, such as fiber degeneration or damage to the recurrent laryngeal nerve, resulting in vocal cord paralysis [14]. The main indications for surgery reported in the literature are functioning PCs, uncertain diagnosis of PCs, the possibility of malignancy, symptomatic PCs, and recurrences. According to Xu et al., the optimal treatment for nonfunctional PCs with diameters greater than 2.5 cm is surgical resection [15]. In our case, the patient underwent cyst resection without any postoperative complications or recurrence during the follow-up.

## Conclusions

PCs are relatively rare lesions that should be considered in the differential diagnosis of cervical masses. Preoperative diagnosis of PCs is challenging; however, fine-needle aspiration and ﬂuid PTH measurement offer a valuable diagnostic tool. Most cases are diagnosed intraoperatively or postoperatively via the pathological analysis of the surgical specimen. Surgical resection is still the optimal treatment for these cysts.
